# An end-to-end pancreatic anastomosis in robotic central pancreatectomy

**DOI:** 10.1186/s12957-019-1609-5

**Published:** 2019-04-13

**Authors:** Zi-Zheng Wang, Guo-Dong Zhao, Zhi-Ming Zhao, Yuan-Xing Gao, Yong Xu, Zhu-Zeng Yin, Qu Liu, Wan Yee Lau, Rong Liu

**Affiliations:** 1 Second Department of Hepatopancreatobiliary Surgery, Chinese People’s Liberation Army (PLA) General Hospital, 28 Fuxing Road, Beijing, 100853 China; 2 Faculty of Medicine, The Chinese University of Hong Kong, Prince of Wales Hospital, Shatin, New Territories Hong Kong

**Keywords:** Robotic surgery, Central pancreatectomy, End-to-end pancreatic anastomosis

## Abstract

**Background:**

Suturing the proximal pancreatic stump and performing pancreaticoenterostomy for the distal pancreatic stump following central pancreatectomy is a conventional procedure. This reconstruction after resection of the pathological pancreatic lesion brings changes in anatomy and physiology. In this study, an innovative one-stage robotic end-to-end pancreatic anastomosis was reported to replace the conventional pancreaticoenterostomy following central pancreatectomy.

**Materials and methods:**

The clinical data of 11 consecutive patients who underwent robotic central pancreatectomy with end-to-end pancreatic anastomosis between August 2017 and December 2017 were analyzed retrospectively.

**Results:**

All operations were completed successfully without any conversion to open surgery. Nine patients had benign tumors, one had a mass-forming chronic pancreatitis, and one had an isolated pancreatic metastasis from a renal cancer. The mean gap left after central pancreatectomy was 4.3 ± 1.0 cm. The median operative time was 121 (range, 105 to 199) min. The median blood loss was 50 (range, 20 to 100) ml. Seven (63.6%) patients developed complications which included Clavien–Dindo Grade I complications in five patients, a Grade II complication in one patient, and a Grade IIIa complication in one patient. Seven patients developed a Grade B postoperative pancreatic fistula, and two patients a biochemical leak. There was no Grade C or worse pancreatic fistula. Magnetic resonance cholangiopancreatography at postoperative 6 months showed no stricture in any of the main pancreatic ducts. Three patients had an asymptomatic and small pancreatic pseudocyst.

**Conclusion:**

Robotic central pancreatectomy with end-to-end pancreatic anastomosis was safe and feasible. It restores the normal anatomy of the pancreas. With its good short-and long-term outcomes, it could be an alternative reconstructive method to pancreaticoenterostomy following central pancreatectomy.

## Introduction

Various operative approaches have been attempted to resect pathological lesions in the neck and body of the pancreas [1, 2]. For benign and low malignant potential lesions in these regions, central pancreatectomy is commonly used, whereas more aggressive resections, such as pancreaticoduodenectomy or distal pancreatectomy, are usually used for malignant lesions [3, 4]. As early as the 1900s, Ehrhardt and Finney reported on resection of the central portion of the pancreas, followed by reconstruction of the pancreas by direct suturing of the two pancreatic stumps [5]. The medical literature became completely silent on central pancreatectomy followed by reconstruction of the pancreas for 70 or more years. In 1982, Dagradi and Serio reported on central pancreatectomy followed by reconstruction of the pancreas by oversewing the cephalic stump and performing an end-to-end pancreaticojejunostomy for the distal pancreatic stump [6]. Since then, this method of reconstruction became the conventional procedure following open, laparoscopic, and robotic central pancreatectomy [7–9].

Central pancreatectomy is a parenchyma-sparing surgery which conserves the exocrine and endocrine functions of the pancreas. There are inherent defects in the conventional reconstructive procedure. For pancreaticojejunostomy of the distal pancreatic stump, a Roux-en-Y limb of jejunum should be created and then delivered through an incision in the transverse mesocolon for the anastomosis, thus affecting the continuity and integrity of the small intestine [10, 11]. The jejunal juice which contains bile can activate pancreatic enzymes from the distal pancreas, leading to erosion of the anastomosis, bleeding, and fistula [12]. The use of pancreaticogastrostomy is an attractive alternative to pancreaticojejunostomy. This procedure is technically easy and safe, as the stomach is close to the pancreatic stump and it has an abundant blood supply [13, 14]. There is, however, a potential harmful effect on the exocrine function of the pancreas, as acid gastric juice inactivates pancreatic enzymes [15, 16].

In the recent one to two decades, only anecdotal reports were published on the end-to-end anastomosis of the pancreas following central pancreatectomy, even though this reconstructive technique is straightforward and accords with normal physiology and anatomy [17–19]. This can partly be explained by the suboptimal anastomotic techniques and the limited operative views in the past.

In the past one to two decades, minimally invasive pancreatic surgery has undergone fast development [20–23]. Minimally invasive equipment and instruments allow surgeons to perform operations with less trauma. The robotic surgical system overcomes several drawbacks of the laparoscopic system and allows more complex procedures to be carried out [24–26]. In this study, our initial clinical experience on robotic end-to-end pancreatic anastomosis following central pancreatectomy was reported [27].

## Materials and methods

### Patients

**Fig. 1:**
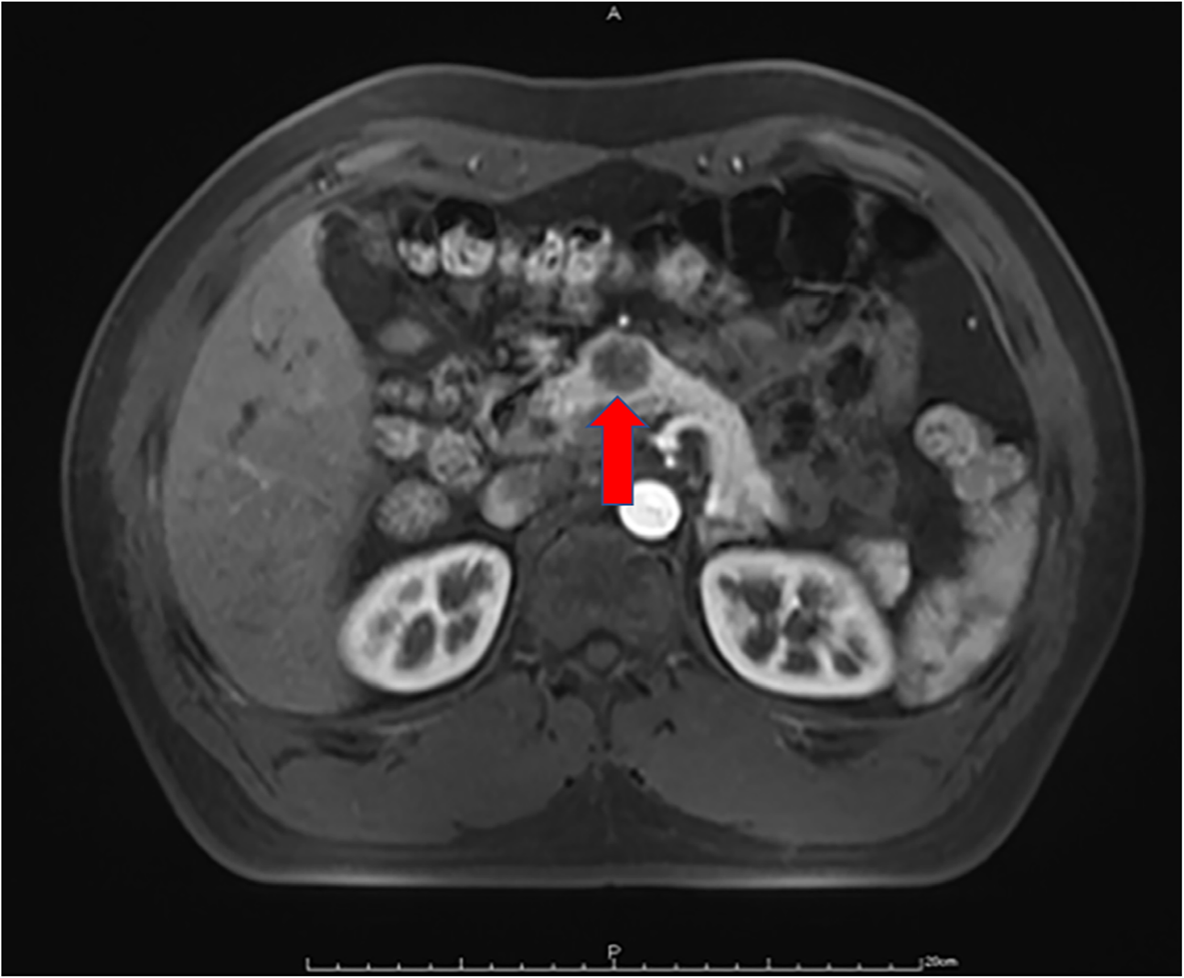
Preoperative MRI showed a tumor in the pancreatic neck (red solid arrow)

From August 2017 to December 2017, consecutive patients who met the inclusion criteria were treated with robotic central pancreatectomy at the Second Department of Hepatopancreatobiliary Surgery, the Chinese People’s Liberation Army (PLA) General Hospital. The inclusion criteria were (1) benign lesions and tumors with low malignant potentials, (2) tumors located in the pancreatic neck and proximal body, (3) tumors close to or had invaded the main pancreatic duct and were not suitable for enucleation, and (4) an estimated defect of the main pancreatic duct ≤ 5 cm after central pancreatectomy (Fig. 1). Patients with suspected pancreatic malignancies or a distal pancreatic stump shorter than 5 cm were excluded. All the operations were performed by a single surgical team. Endoscopic ultrasonography, CT, MRI, or PET-CT were done preoperatively for diagnosis and assessment. The patients’ demographic data, clinicopathological characteristics, and perioperative outcomes were retrospectively reviewed. The study was conducted in accordance with the ethical principles of the Helsinki Declaration for research on humans. The study was approved by the Ethical Committee of the PLA Central Hospital. Written informed consent was obtained from the all individual participants included in the study.

### Surgical techniques

**Fig. 2:**
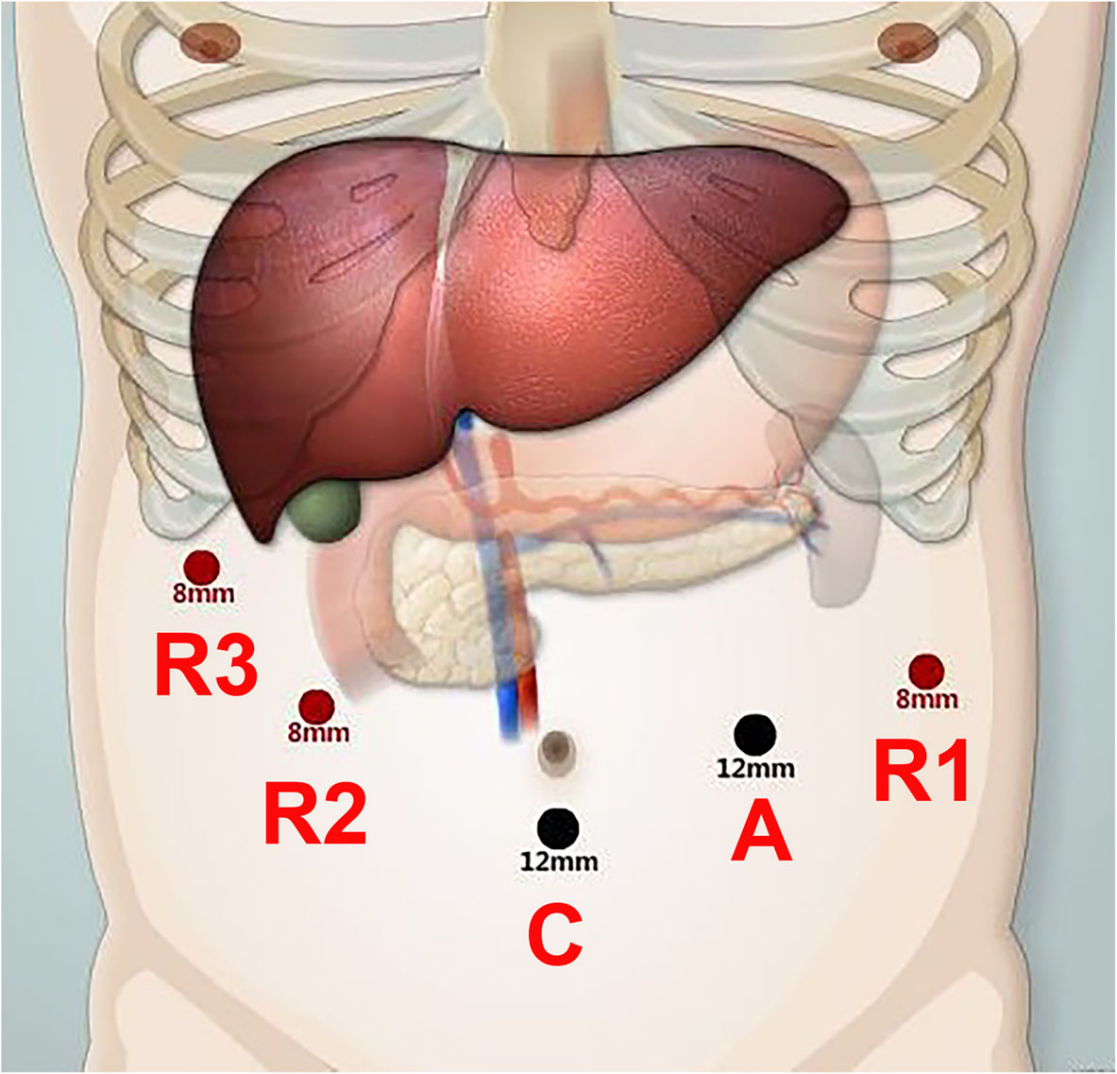
Ports placement in robotic central pancreatectomy. C: camera port, **a** assistant port, R1–R3: ports for robotic arms

All surgeries were completed by the Si model of the da Vinci robotic surgical system (Intuitive Surgical, Inc., Sunnyvale, CA) by experienced hepatopancreatobiliary surgical team that has accomplished more than 1500 cases of robotic pancreatic surgery. Patients were placed in a supine position with a pad to lift up the left loin. After pneumoperitoneum was established, five trocars were utilized, similar to those used for robotic distal pancreatectomy (Fig. 2). The camera port (C) was created below the umbilicus, the assistant port (A) at the lower left of the umbilicus, the port for the first robotic arm (R1) in the left anterior axillary line at the level of umbilicus, the port for the second robotic arm (R2) in the right mid-clavicular line at the level of the umbilicus (to be used with the method of “Trocar in Trocar”), and the port (8 mm) for the third robotic arm (R3) under the costal margin in the right middle axillary line.

**Fig. 3:**
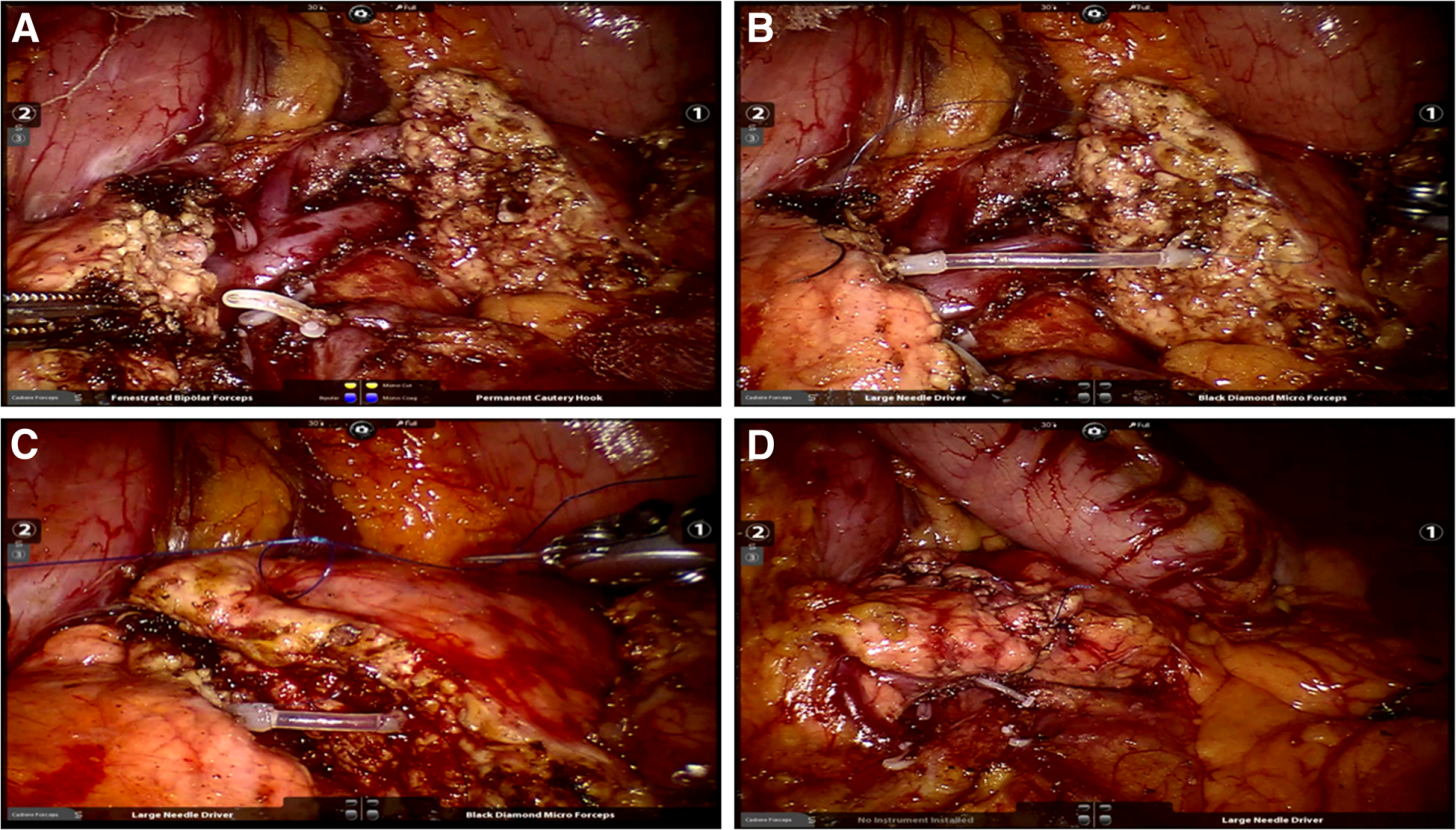
Intraoperative pictures. **a** Completion of central pancreatectomy. **b** Insertion of the pancreatic ductal stent. **c** End-to-end anastomosis of the two pancreatic stumps. **d** Completion of end-to-end anastomosis

**Fig. 4:**
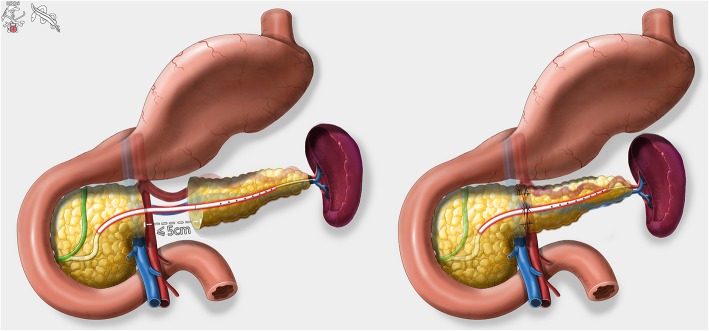
Animation of end-to-end pancreatic anastomosis

The gastrocolic ligament was opened to enter the lesser sac to expose the anterior surface of the pancreas. The tumor was located with laparoscopic ultrasonography and the resection planes were determined. The superior and inferior borders of the pancreatic neck and proximal body were carefully exposed. A tunnel between the posterior of the pancreatic neck and the portal/superior mesenteric/splenic veins was progressively created. Along the planned transection plane away from the pathological lesion, the pancreatic parenchyma was transected with an ultrasonic scalpel and the main pancreatic duct with scissor sharply. The central pancreas together with the lesion was resected. The two stumps of the main pancreatic duct were identified. A proper sized pancreatic stent (of 5–10 cm long) was chosen and gently inserted into the two pancreatic ductal stumps. The pancreatic stent was fixed to the distal ductal stump using a single stitch of an absorbable suture (5–0 PDS-II, Ethicon, USA). Then, for the reinforcement and hemostasis of the two pancreatic stumps, the inferior and superior portions of the two stumps were oversewn with a vertical figure of 8 suture, and the middle portion with U-shaped sutures (4–0 Prolene, Ethicon, USA). The proximal and distal pancreatic stumps were then further dissected with an aim to facilitate a subsequent tension-free anastomosis. Since most of lesions were benign and borderline, the main pancreatic duct was not dilated and too thin to perform a precise duct-to-duct anastomosis. The cephalic and caudal stumps of pancreas were then pulled together directly. Anastomosis of the posterior portion of the pancreatic stumps was carried out using a continuous suture (4–0 Prolene, Ethicon, USA). The anterior portion of the pancreatic stumps was then anastomosed by a continuous suture (4–0 Prolene, Ethicon, USA) (Figs. 3 and 4). Two drains were placed at the superior and inferior borders of the pancreas and extracted through the R2 port. The specimen was placed in an endo-bag, and extracted through the enlarged umbilical vertical incision of the camera port.

### Postoperative care

The patients were closely monitored for vital signs for about 24 h postoperatively. Antibiotics, somatostatin, proton pump inhibitors, and parenteral nutrition were routinely given. The patients were prescribed adequate analgesia and they were encouraged to have early mobilization. The nasogastric tube was typically removed on postoperative day 1. The drain outputs were carefully monitored for the volumes and amylase levels. The drain amylase level and bacteria culture were routinely tested on postoperative day 3. The drains were removed if the drainage was less than 5 ml per day with a low amylase level.

### Statistical analysis

The demographic data, clinicopathological characteristics, and perioperative outcomes were presented as frequency for categorical variables, and mean ± standard deviations or median (range), as appropriate, for continuous variables based on normality.

## Results

From August 2017 to December 2017, 11 patients underwent robotic central pancreatectomy followed by end-to-end pancreatic anastomosis. There was no conversion to open surgery. The clinicopathological features and perioperative outcomes of the patients are shown in Table 1 and Table 2, respectively. There were six male and five female patients. The mean age was 42.4 ± 14.3 years. The mean BMI was 24.1 ± 2.8 kg/m^2^. Ten patients were ASA Grade II and 1 Grade I. The pancreatic duct was not dilated in all the patients. The mean diameter of the pancreatic duct was 2.4 ± 0.3 mm. The mean diameter of the lesions was 3.4 ± 1.1 cm. The mean gap of the pancreas left after central pancreatectomy was 4.3 ± 1.0 cm. The median operative time was 121 (range, 105 to 199) min. The median estimated blood loss was 50 (range, 20 to 100) ml.

**Table 1:** The clinicopathological characteristics of patients

Baseline and intraoperative data	
Sex (female/male), *n*	5/6
Age, mean ± SD (years)	42.4 ± 14.3
BMI, mean ± SD (kg/m^2^)	24.1 ± 2.8
ASA(I/II), *n*	1/10
Tumor size, mean ± SD (cm)	3.4 ± 1.1
Pancreatic duct diameter, mean ± SD (mm)	2.4 ± 0.3
Defect of main pancreatic duct, mean ± SD (cm)	4.3 ± 1.0
Operative time, [median (range)] (min)	121 (105, 199)
Estimated blood loss, [median (range)] (ml)	50 (20, 100)
Open conversion, *n* (%)	0 (0)
Pathology	
Solid pseudopapillary tumor, *n* (%)	6 (54.5)
Serous cystadenoma, n (%)	3 (27.3)
Pancreatic metastasis from renal cancer, *n* (%)	1 (0.09)
Mass-forming chronic pancreatitis, *n* (%)	1 (0.09)
Negative margin in tumor (*n*)	10 (100%)

**Table 2:** The short-term and long-term outcomes of patients

Short-term outcomes	
Postoperative hospital stays, [median (range)] (day)	6 (5–9)
Complication, *n* (%) [1]	7 (65.6)
Clavien–DindoI/II/IIIa, *n* (%)	5 (45.5)/1 (9.1)/1 (9.1)
Grade B pancreatic fistula, *n* (%)^a^	7 (65.6)
Peripancreatic fluid collection, *n* (%)	1 (9.1)
Postoperative pancreatitis, *n* (%)	1 (9.1)
Drain removal time, mean ± SD (day)	36.3 ± 16.8
30-day readmission, *n* (%)	0 (0)
90-day mortality, *n* (%)	0 (0)
Long-term outcomes	
Follow-up period, [median (range)] (month)	11.7(8.1–12.2)
Spontaneous detachment of pancreatic stent, *n* (%)	11 (100)
Pancreatic anastomosis stricture, *n* (%)	0 (0)
Pancreatic pseudocyst and discontinuous main pancreatic duct, *n* (%)	3 (27.3%)
Postoperative diabetes, *n* (%)	0 (0)

^a^Based on the 2016 update of the International Study Group (ISGPS) definition and grading of postoperative pancreatic fistula

**Fig. 5:**
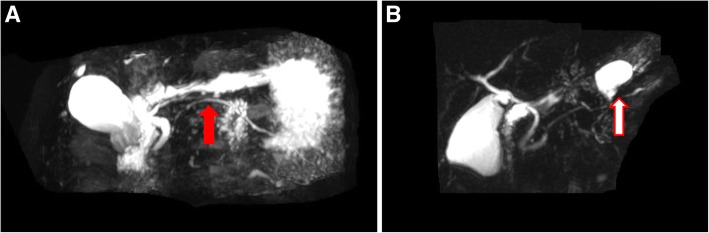
**a** Postoperative MRCP showed a good continuity in the main pancreatic duct (red solid arrow). **b** Postoperative MRCP showed a disconnection in the main pancreatic duct with development of a pancreatic pseudocyst at the reconstruction site (red hollow arrow)

Histopathological examination demonstrated that there were solid pseudopapillary tumors in six patients, serous cystadenomas in three patients, a solitary pancreatic metastasis from renal cancer in one patient, and a mass-forming chronic pancreatitis in one patient. All the resections were achieved with a negative resection margin. The median postoperative hospital stay was 6 (range, 5–9) days. Seven (63.6%) patients developed complications which included Clavien–Dindo Grade I complications in five patients, a Grade II complication in one patient, and a Grade IIIa complication in one patient. Based on the 2016 update of the International Study Group (ISGPS) definition and grading of postoperative pancreatic fistula (POPF) [28], two patients had biochemical leak and seven patients had Grade B POPF. One patient with a Grade B POPF was treated with ultrasound-guided drainage for peripancreatic fluid collection. The remaining patients, including a patient with postoperative acute pancreatitis, recovered with conservative treatment. The median follow-up period was 15.9 (range, 12.2–16.0) months. Magnetic resonance cholangiopancreatography (MRCP) at postoperative 6 months showed eight patients had good continuity in the main pancreatic ducts. However, in three patients, there was a disconnection in the main pancreatic ducts with development of a pancreatic pseudocyst at the reconstruction site (Fig. 5). All the pancreatic stents passed out of the patient’s bodies spontaneously within 6 months after surgery. All the patients were on a normal diet with no abdominal symptoms at the last follow-up.

## Discussion

The most commonly performed radical resections for pancreatic malignancies are pancreaticoduodenectomy and distal pancreatectomy +/− splenectomy [29]. However, benign lesions and tumors of low malignant potential do not require extensive resections [4], and pancreatic enucleation and central pancreatectomy are adequate to treat these lesions [30, 31]. When these lesions are superficial in the pancreas, enucleation can achieve good outcomes. When these lesions are deeply situated in the pancreas or when they are close to the main pancreatic duct, enucleation may damage the duct. Even with successful repair of a damaged duct which technically is very difficult, POPF is likely to occur [32]. In the past two decades, central pancreatectomy has been increasingly used to treat benign lesions and tumors with low malignant potentials in the central pancreas. This operation conserves more pancreatic parenchyma but the conventional reconstruction using pancreaticoenterostomy disrupts the continuity and integrity of the small intestine which can potentially lead to short- and long-term complications [7, 9].

The end-to-end anastomosis of the pancreatic stumps after central pancreatectomy has all along been considered by mainstream pancreatic surgeons to be unreliable [1], and prone to result in severe POPF, especially when the texture of pancreas is normal and the main pancreatic duct is not dilated. In the past, the end-to-end anastomosis of the pancreas has been used to repair traumatic pancreatic neck transections. For the various techniques which have been reported, one technique was to use a pancreatic stent and to perform an end-to-end anastomosis of the pancreatic duct and parenchyma [33]. Another technique was to do the pancreatic anastomosis using a stent put into the pancreatic duct which was then brought through the ampulla of Vater, through the duodenum into the stomach, and then exteriorized through a gastrostomy [34]. Other techniques include pancreatic duct opposition with or without ductal anastomosis, and with or without pancreatic parenchymal anastomosis [35]. The postoperative complications and long-term follow-up of these reported cases were favorable. In pancreatic trauma, unlike in central pancreatectomy, the gap left between the two pancreatic stumps is much less. The earliest report on the use of the end-to-end anastomosis following central pancreatectomy was in the 1900s. This operation was seldom used subsequently [5]. An experimental study in dogs using an end-to-end anastomosis with or without stenting following central pancreatectomy suggested that this reconstructive technique was practicable [36]. Subsequently, only occasional case reports on one to three patients using this technique for pancreatic reconstruction after central pancreatectomy were reported [17–19]. These reports routinely exteriorized a stent through the ampulla of Vater for internal-or-external drainage. Ramesh [17] added a serosal patch from a Roux-en-Y limb of the jejunum to the anterior suture line to buttress the anastomosis.

The robotic surgical system is an upgraded surgical platform of the traditional laparoscopic system over which it has several virtues which include the flexible Endo-wrist instruments, tremor elimination, 3D magnified view, as well as persistent and stable traction by the robotic arm. These advantages of the robotic surgical system enable operative procedures to become more delicate and precise, particularly for dissection and anastomosis of tiny vasculatures. The key technique in the end-to-end anastomosis in our operation is the need to fully mobilize the distal pancreatic stump by transecting the peripancreatic ligaments. The main pancreatic duct of most patients is not dilated and too thin to perform a precise duct-to-duct anastomosis. The parenchyma of the pancreatic head and tail are then pulled together to approximate the two pancreatic duct stumps. MRCP at postoperative 6 months indicated that the “pull-together” approach had good effect and no stricture of the main pancreatic duct happened. The limitations of this study are the small case number and the inherent defects of its retrospective study nature. In the future research, studies such as randomized controlled trial, propensity score matching study to compare this technique to conventional technique with larger cohort are needed to further define the efficacy of this technique.

In this cohort, 65.6% (7/11) of the patients developed Grade B pancreatic fistula because of a persistent drainage > 3 weeks, including a patient with a peripancreatic fluid collection and a patient with postoperative acute pancreatitis. We think the high postoperative pancreatic fistula rate might be associated with the soft texture of the pancreas without malignancy in this cohort. It also may be attributed to our preliminary experience. Despite the POPF rate is high, most of the patients with POPF only have a prolonged drainage without and recovered uneventfully without other complications.

## Conclusion

Robotic central pancreatectomy with end-to-end pancreatic anastomosis allowed resection of lesions with the least injury, maximized preservation of pancreatic parenchyma, and maintained normal anatomy and physiology after surgery. Our preliminary clinical experience suggested that this end-to-end anastomosis following robotic central pancreatectomy was safe and feasible. Although the POPF rate is high, most of the patients with POPF only have a prolonged drainage without clinical relevant change in the management of POPF. It could be used as an alternative to pancreaticoenterostomy following central pancreatectomy. Comparative study with larger cohort and further modification of the technique are needed to define the efficacy of this technique.
